# Vision-based markerless registration using stereo vision and an augmented reality surgical navigation system: a pilot study

**DOI:** 10.1186/s12880-015-0089-5

**Published:** 2015-11-02

**Authors:** Hideyuki Suenaga, Huy Hoang Tran, Hongen Liao, Ken Masamune, Takeyoshi Dohi, Kazuto Hoshi, Tsuyoshi Takato

**Affiliations:** Department of Oral-Maxillofacial Surgery, Dentistry and Orthodontics, The University of Tokyo Hospital, 7-3-1 Hongo, Bunkyo ku, Tokyo, 113 8656 Japan; Department of Mechano-Informatics, Graduate School of Information Science and Technology, The University of Tokyo, Tokyo, Japan; Department of Bioengineering, Graduate School of Engineering, The University of Tokyo, Tokyo, Japan; Department of Biomedical Engineering, School of Medicine, Tsinghua University, Beijing, China; Faculty of Advanced Technology and Surgery, Institute of Advanced Biomedical Engineering and Science, Tokyo Women’s Medical University, Tokyo, Japan; Department of Mechanical Engineering, School of Engineering, Tokyo Denki University, Tokyo, Japan

**Keywords:** Augmented reality, Integral videography, Markerless registration, Stereo vision, Three-dimensional image

## Abstract

**Background:**

This study evaluated the use of an augmented reality navigation system that provides a markerless registration system using stereo vision in oral and maxillofacial surgery.

**Method:**

A feasibility study was performed on a subject, wherein a stereo camera was used for tracking and markerless registration. The computed tomography data obtained from the volunteer was used to create an integral videography image and a 3-dimensional rapid prototype model of the jaw. The overlay of the subject’s anatomic site and its 3D-IV image were displayed in real space using a 3D-AR display. Extraction of characteristic points and teeth matching were done using parallax images from two stereo cameras for patient-image registration.

**Results:**

Accurate registration of the volunteer’s anatomy with IV stereoscopic images via image matching was done using the fully automated markerless system, which recognized the incisal edges of the teeth and captured information pertaining to their position with an average target registration error of < 1 mm. These 3D-CT images were then displayed in real space with high accuracy using AR. Even when the viewing position was changed, the 3D images could be observed as if they were floating in real space without using special glasses.

**Conclusion:**

Teeth were successfully used for registration via 3D image (contour) matching. This system, without using references or fiducial markers, displayed 3D-CT images in real space with high accuracy. The system provided real-time markerless registration and 3D image matching via stereo vision, which, combined with AR, could have significant clinical applications.

**Electronic supplementary material:**

The online version of this article (doi:10.1186/s12880-015-0089-5) contains supplementary material, which is available to authorized users.

## Background

Augmented reality (AR) involves the co-display of a virtual image and a real-time image so that the user is able to utilize and interact with the components of both images simultaneously [1]. This image-based navigation facilitates *in situ* visualization during surgical procedures [2] because visual cues obtained from a preoperative radiological virtual image can enhance visualization of surgical anatomy [3], thus improving preoperative planning and supporting the surgeon’s skill by simplifying the anatomical approach to complex procedures [4]. In recent years, the technical application of AR has been studied in the context of various clinical applications. Examples of recent research which has sought to determine how the application of AR may lead to improvements in medical outcomes have included a study examining the use of AR to improve the precision of minimally invasive laparoscopic surgeries [3]; comparison between planned and actual needle locations in MRI-guided lumbar spinal injection procedures [5]; and studies examining the application of AR for image-guided neurosurgery for brain tumors [6], for the overlay of preoperative radiological 3-dimensional (3D) models onto the intraoperative laparoscopic videos [7]; and to facilitate vessel localization in neurovascular surgery [8]. AR also has potential as an aid to surgical teaching [4]. Furthermore, CT-free navigation systems, which do not rely on the acquisition of pre-procedure image acquisition but instead intra-operatively recognize the position and orientation of defined patient features, are also being evaluated [9, 10]. AR has the potential to increase the surgeon’s visual awareness of high-risk surgical targets [7] and to improve the surgeon’s intuitive grasp of the structures within the operational fields [11].

Similarly, there are an increasing number of studies examining the potential use of image-guided systems for oral and maxillofacial surgeries (OMS) [12, 13]. Patient or image registration (overlay) is key to associating the surgical field with its virtual counterpart [14, 15]. The disadvantages of the current navigation systems used in OMS include bulky optical trackers and lower accuracy of electromagnetic trackers in locating surgical instruments, invasive and error-prone image registration procedures, and an additional reference marker to track patient movement [16]. In addition, errors related to position, angle, distance, vibration of the optical tracker, the reference frame and probe tip of the equipment are high. With anatomical landmark-based registration, each observer is only prone to human error based on personal preference of anatomical landmarks in the surgical field [17, 18]. Moreover, frequent hand-eye transformation, which corrects the displacement between the probe tip and the image reference frame, is required for constant comparisons between the surgical field and the displayed image. Furthermore, images in real space are projected using a 3D display via binocular stereopsis, with the disadvantage that the observed video does not change with changes in viewing position since only relative depth is recognized. Thus, accurate 3D positioning cannot be reproduced without incurring motion parallax. Head mounted displays and head-mounted operating microscopes with stereoscopic vision have been used many times for AR visualization in the medical field. However, such video see through devices have two views that present only horizontal parallax, instead of the full parallax. Projector-based AR visualization is appropriate for large operative field overlays; however, it lacks depth perception. As described in our previous study, we have developed an autostereoscopic 3-D image overlay using a translucent mirror [15]. The integral videography (IV) principle applied in this study differs from binocular stereopsis, and allows both binocular parallaxes for depth perception and motion parallax, wherein depth cues are recognized even if the observer is in motion [15, 19–21]. Results from our previous research have shown that the 3D AR system using integral video-graphic images is a highly effective and accurate tool for surgical navigation in OMS [22].

To overcome the challenges of image-guided OMS, we developed a more simplified AR navigation system that provides automatic markerless image registration using real-time autostereoscopic 3D (IV) imaging and stereo vision for dental surgery. Patient-image registration achieved by patient tracking via contour matching has been previously described [14]. The current study evaluated the feasibility of using a combination of AR and stereo vision technologies to project IV images obtained from preoperative CT data onto the actual surgical site during real time and automatic markerless registration, respectively, in a clinical setting; this feasibility study was performed on a volunteer. Therefore, this study proposes use of this simplified image-guided AR technology for superimposing a region-specific 3D image of the jaw bone on the actual surgical site in real time. This technology can aid surgical treatment of structures that are in spatial positions but not directly observable.

## Methods

**Fig. 1:**
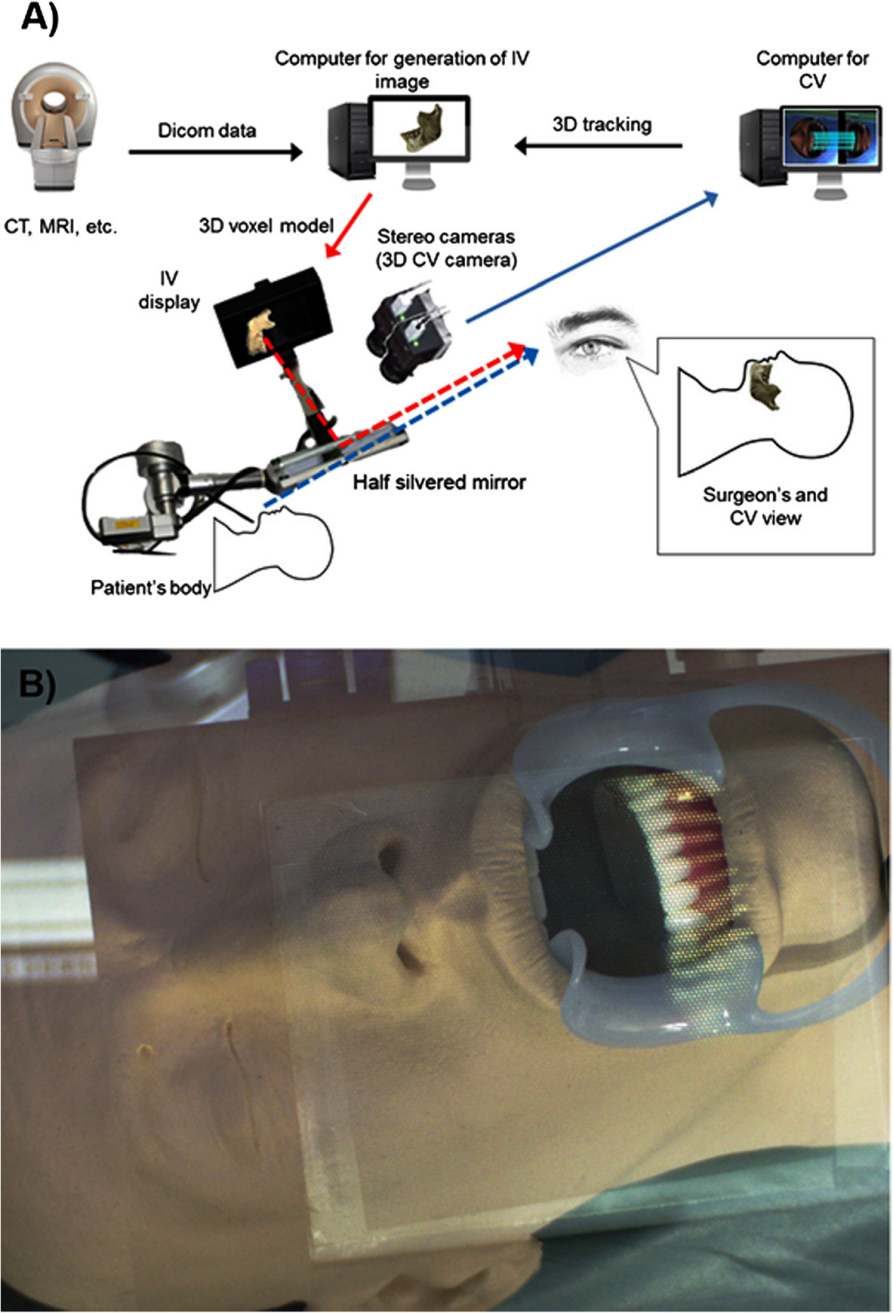
The physical setup of the system. **a** The configuration of the markerless surgical navigation system based on stereo vision and augmented reality, and **b** a 3D rapid prototyping model

The apparatus for the entire system was comprised of 3D stereo camera and the 3D-IV imaging system, as shown in Fig. 1a. We used two computer systems; one to track the surgical procedure using stereo vision and the other to generate 3D-IV images for a projected overlay. The study was conducted in accordance with Good Clinical Practice (GCP) guidelines and the Declaration of Helsinki, and the study protocol was approved by the medical ethics committee of the Graduate School of Medicine of the University of Tokyo, Japan. Written informed consent was provided by the volunteer prior to study initiation.

### Generation of 3D-IV images

The 3D-IV images to be projected onto the surgical site were generated from CT images of the jaw bones using an Aquilion ONE™ (Toshiba, Tokyo, Japan) 320-row area detector CT scanner and from images of the teeth using a Rexcan DS2 3D scanner (Solutionix, Seoul, Korea). Conditions for the area detector CT (ADCT) scan were: tube voltage, 120 kV; tube current, 270 mA; and slice thickness, 0.5 mm. Thus, the IV image generated from the preoperative CT data was a “real” 3D representation of the jaws. Next, 3D surface models of the upper and lower jawbones were generated using Mimics® Version 16 (Materialise, Leuven, Belgium) and Geomagic Control (Geomagic, Cary, NC, USA) medical image-processing software. Similarly, the 3D scanned images of a dental plaster model were recorded onto a CT image using the Rexcan DS2 3D scanner. Briefly, the IV image of the 3D CT was constructed as an assembly of reconstructed light sources. The complete 3D-IV image was displayed directly onto the surgical site using a half-silvered mirror (Fig. 1a), which makes it appear that the 3D image is inside the patient's body, and could be viewed directly without special glasses. Technical details for the generation of the 3D-IV images have been described in previous publications [15, 19, 20, 22, 23]. Each point, shown in a 3D space, was reconstructed at the same position as the actual object by the convergence of rays from the pixels of the element images on the computer display after they pass through the lenses in a microconvex lens array. The surgeon can see any point on the display from various directions, as though it were fixed in 3D space. Each point appears as a different light source. The system was able to render IV images at a rate of 5 frames per second. For the IV image to be displayed in the correct position, the coordinates of the preoperative model obtained from CT data are registered intra-operatively with the contour derived from the 3D scanner image of the subject in real space. The triangle mesh model of the teeth is created using the marching cubes algorithm and is rendered by OpenGL.

### Rapid prototyping model

With the 3D-IV images generated, a feasibility study was conducted on a phantom head using a 3D rapid prototyping (RP) model of the mandible using Alaris™ 30U RP technology (Objet Geometries, Rehovot, Israel) based on CT data of the subject (Fig. 1b). Technical details of the registration of the RP model have been described in previous publications, with registration errors reported to be between 0.27 and 0.33 mm [18]. The mean overall error of the 3D image overlay in the current study was 0.71 mm, which met clinical requirements [15].

### Patient tracking

“Patient tracking” refers to tracking of the 3D contours of the teeth (incisal margins). The incisal margins were tracked, with the right and left images obtained through the stereo camera in real time; spatial positions of the teeth were obtained by matching the right and left images for 3D-contour reconstruction. The reconstructed 3D image was then compared with the actual image from the subject using the stereo camera.

Specifications for the tracking system included an Intel® Core™ i7 3.33 GHz processor combined with an NVIDIA® GeForce® GTX 285 GPU and an EO-0813CCD 2 charge-coupled device stereo camera (Edmund Optics Inc., Barrington, NJ, USA). The camera had a maximum frame rate of 60 frames per second with an image resolution of 1280 × 1024 pixels.

### Image-based calibration of IV display

The 3D-IV images were calibrated using a calibration model with known geometry that included: a) visualization of the calibration model with five feature points in the IV frame; b) display of the 3D image of the calibration model; c) stereo image capture of the 3D image with the stereo camera through the half silvered mirror; and d), matching of parallax images (right and left images) from the stereo camera to obtain a final 3D-IV image. Similarly, the final calibrated 3D-IV images of the subject’s jaw that appeared to be floating in real space were projected into the correct position based on the coordinates of the image obtained from preoperative CT data and the real object from the subject using HALCON software Version 11 (MVTec Software GmbH, Munich, Germany) and Open CV, the Open Source Computer Vision Library.

### Patient-image registration

Typically, fixed external anatomic landmarks on the patient and imaging data define the accuracy of imaging system [24] whereby anatomic landmarks identified on the surface of the organ can be correlated with accuracy to the predefined landmarks in the computer’s coordinate system. In the current study, the natural landmarks (incisal margins) were tracked with the stereo camera instead of manual identification. The 3D position of this natural landmark was accurately determined using the right and left images (parallax images) captured by the stereo camera [25]. Thereafter, the preoperative 3D-CT image was integrated with images from the subject using 3D image-matching technology (stereo vision). The detected feature point of a tooth (actual image) was then correlated with the corresponding feature point of a tooth on the 3D CT image. This means that mutually corresponding feature points in both the images (i.e., 3D-CT image and actual image of a tooth) were matched. Matching of the 3D-CT image and volunteer position was based on the correlation of ≥ 200 feature points on the incisal margin. Because of the high contrast between the teeth and the oral cavity, the 3-D contour of the front teeth is easily extracted using template matching and edge extraction. An image template is first manually selected using the left camera image and then matched to the corresponding right camera image to select the regions of interest (ROI). 2-D edges of the front teeth are then extracted with subpixel accuracy within the detected ROIs, and the extracted teeth edges are stereo-matched using epipolar constraint searching. Sub-pixel estimation is the process of estimating the value of a geometric quantity to better than pixel accuracy, even though the data was originally sampled on an integer pixel quantized space. Frequency based shift calculated methods using phase correlation (PC) have been widely used because of its accuracy and low complexity for shift motion due to translation, rotation or scale changes between images. The PC method for images alignment relies mainly on the shift property of the Fourier transform to estimate the translation between two images. The epipolar line is the straight line of intersection of the epipolar plane with the image plane. It is the image in one camera of a ray through the optical centre and image point in the other camera. All epipolar lines intersect at the epipole. This epipolar line is an extremely important constraint in the stereo matching step. Epipolar constraint searching aims to establish a mapping between points in the left image and lines in the right image and vice versa so that "the correct match must lie on the epipolar line". 11 × 11 area is defined as the patch size of 11 × 11 pixel. A normalized cross correlation coefficient describes the similarity between two patches and can be used for solving correspondence problems between images. The basic steps involves (i) extracting a reference patch from the reference image; the conjugate position of this reference patch in the search image is determined, (ii) defining a search area and specific sampling positions (search patches) for correlation, within the search image, (iii) computing the correlation value (with respect to the reference patch) at each of the defined sample position, and (iv) finding the sample position with the maximum correlation value, which indicates the search patch with highest similarity to the reference patch. If multiple edge points appear on the epipolar line, the one with the closest normalized cross correlation value (calculated in an 11 × 11 area centered at the candidate edge point) is chosen for the match. Full details of the algorithms used for matching have been previously described [15]. An HDC-Z10000 3D video camera (Panasonic Co, Ltd, Tokyo, Japan) was used to document the procedure. Although we adapted the steps from published literature [15, 25], the novelty of our method is that this is the first study in which dentomaxillofacial 3D computed tomography (CT) data (both maxillary and mandibular jaws along with teeth) generated by a 3D IV image display system using augmented reality (AR) navigation system that provides a markerless registration system using stereo vision in oral and maxillofacial surgery were superimposed on a human volunteer. Previous studies were made on phantom models. We focused on investigating the property of the intraoral environment of human. Patient movement was a challenge that needed to be addressed when applying the method on a human subject in real clinical setting. The challenge of patient movement was overcome by the use of a custom designed stereo camera which tracked the patient movement and updated the image registration on real time without manual involvement.

### Evaluation of recognition time and positioning error

Because registration and tracking were performed using the stereo camera, the measurement error of the stereo camera system was considered a major source of registration error. Since it was considered impractical to evaluate the accuracy of each stage of the registration process, the accuracy of the final 3D-IV image was used to confirm the accuracy of this novel system. Error calculation was conducted as per our previous study, based on the alignment of 14 points on the surface of the teeth with the actual 3D-IV images using the stereo camera [23]. Because these points were not used at any stage of the registration process, the accuracy of this experiment can be considered as a target registration error (TRE). Each point was measured 20 times in the stereoscopic model and 3D-IV images to determine average value, standard deviation (SD) and 3D differences for the target position [1]. Calculations were performed according to Fitzpatrick and West [26].

The accuracy of the cameras was based on the following equations:

$$ \begin{array}{l} XYdirection\kern0.24em :\kern0.24em {\varDelta}_x = \kern0.24em {\varDelta}_y=\kern0.36em \frac{z}{f}\kern0.24em {\varDelta}_d\\ {} Zdirection\kern0.24em :\kern0.24em {\varDelta}_z=\frac{z^2}{fb}\kern0.24em {\varDelta}_d\end{array} $$

where, z is the distance from the camera to the object (~500 mm), ƒ is the focal length of the cameras (12 mm), b is the distance between the cameras (120 mm) and ∆_d_ represents half of a pixel’s size on the sensor (2.325 μm).

## Results

**Fig. 2:**
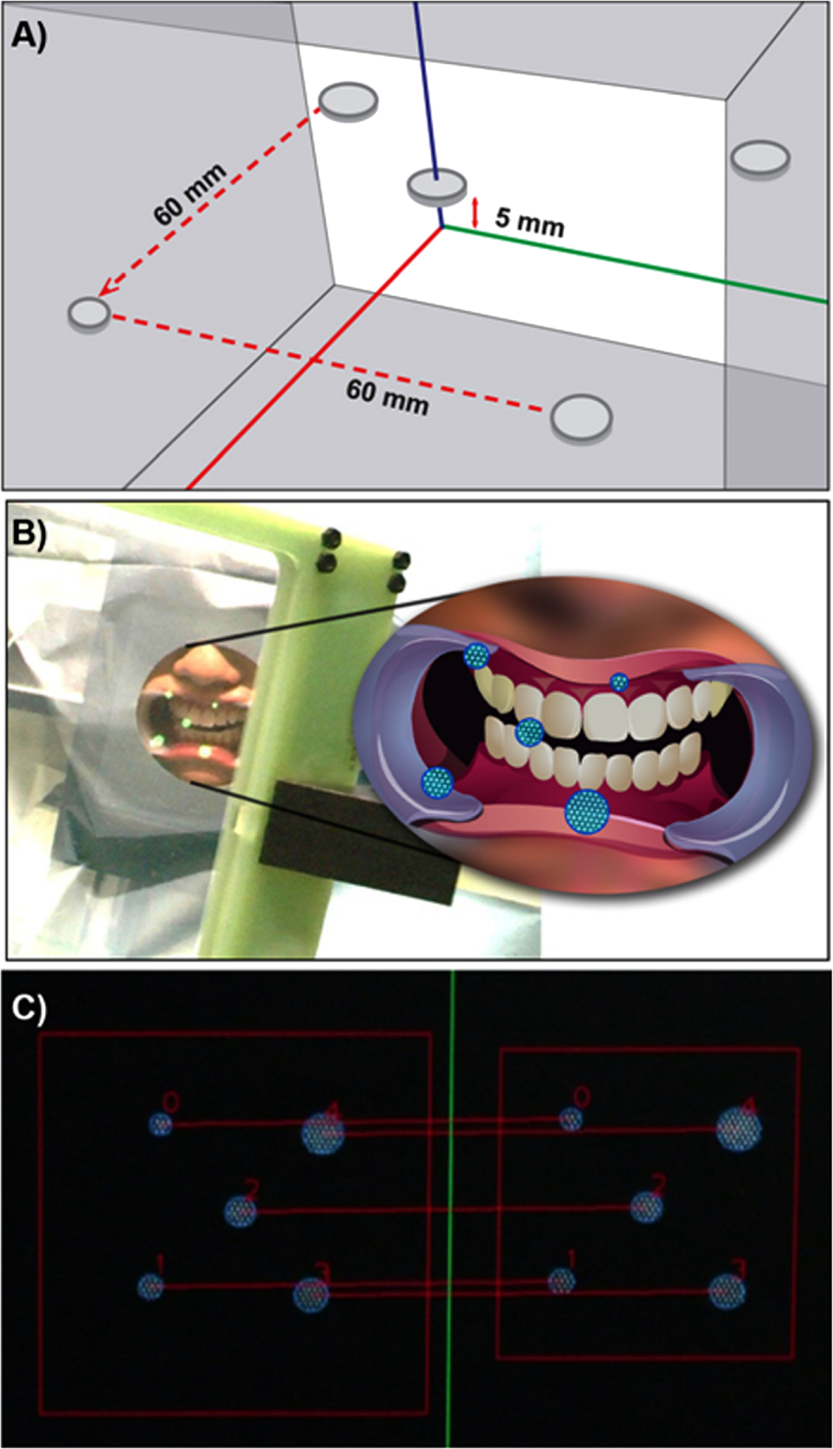
Calibration of the integral videography (IV) display. This includes **a** five feature points for calibration, **b** The IV image displayed in real space, and **c** recognition results for the calibrated IV images (matching of left and right images via stereo vision)

**Fig. 3:**
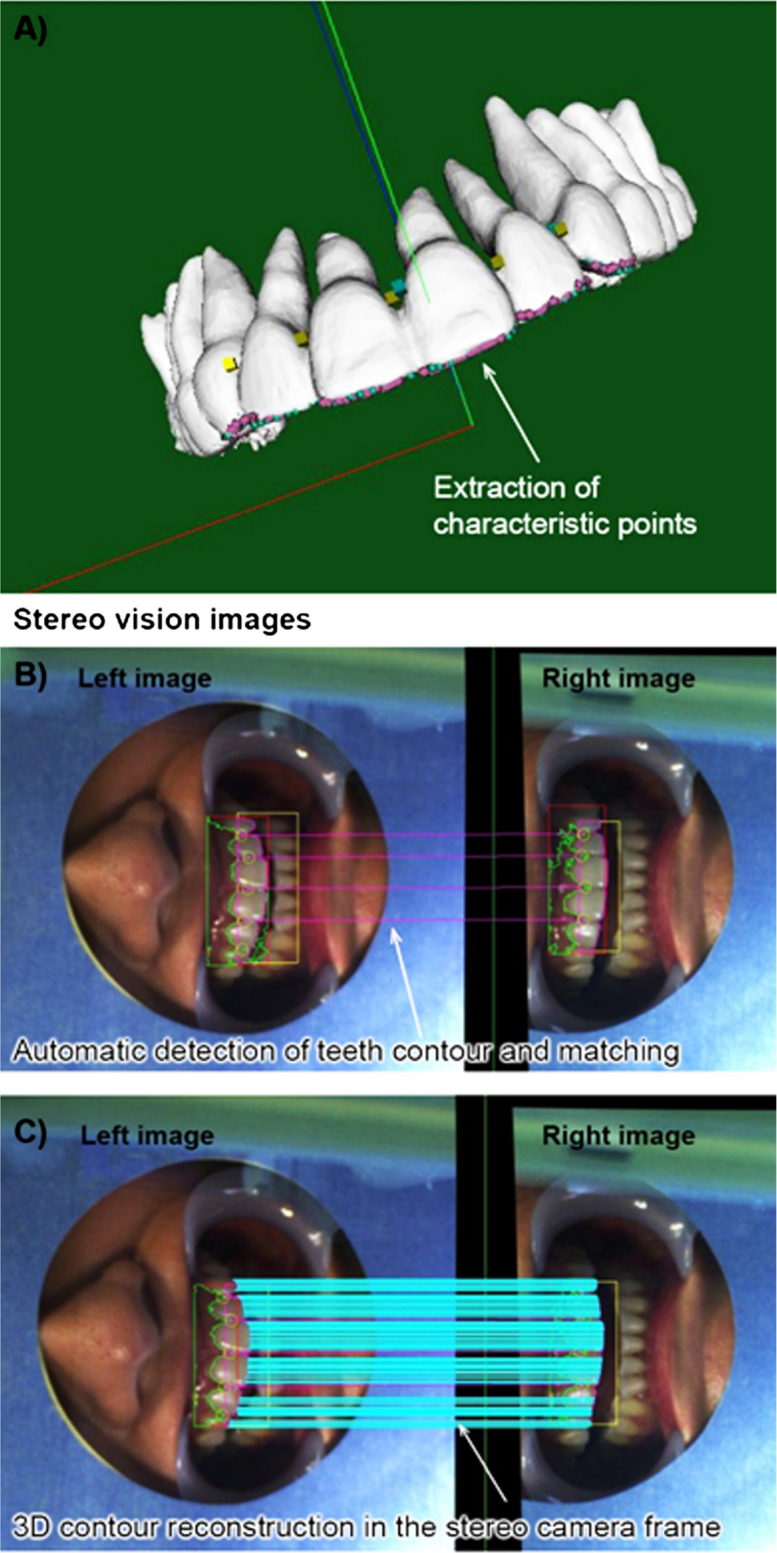
Automatic registration of the 3D-CT image and volunteer’s position. This included **a** extraction of characteristic points, **b** automatic detection of teeth contour and matching of right and left images via stereo vision, and **c** 3D contour reconstruction in the stereo camera frame

**Fig. 4:**
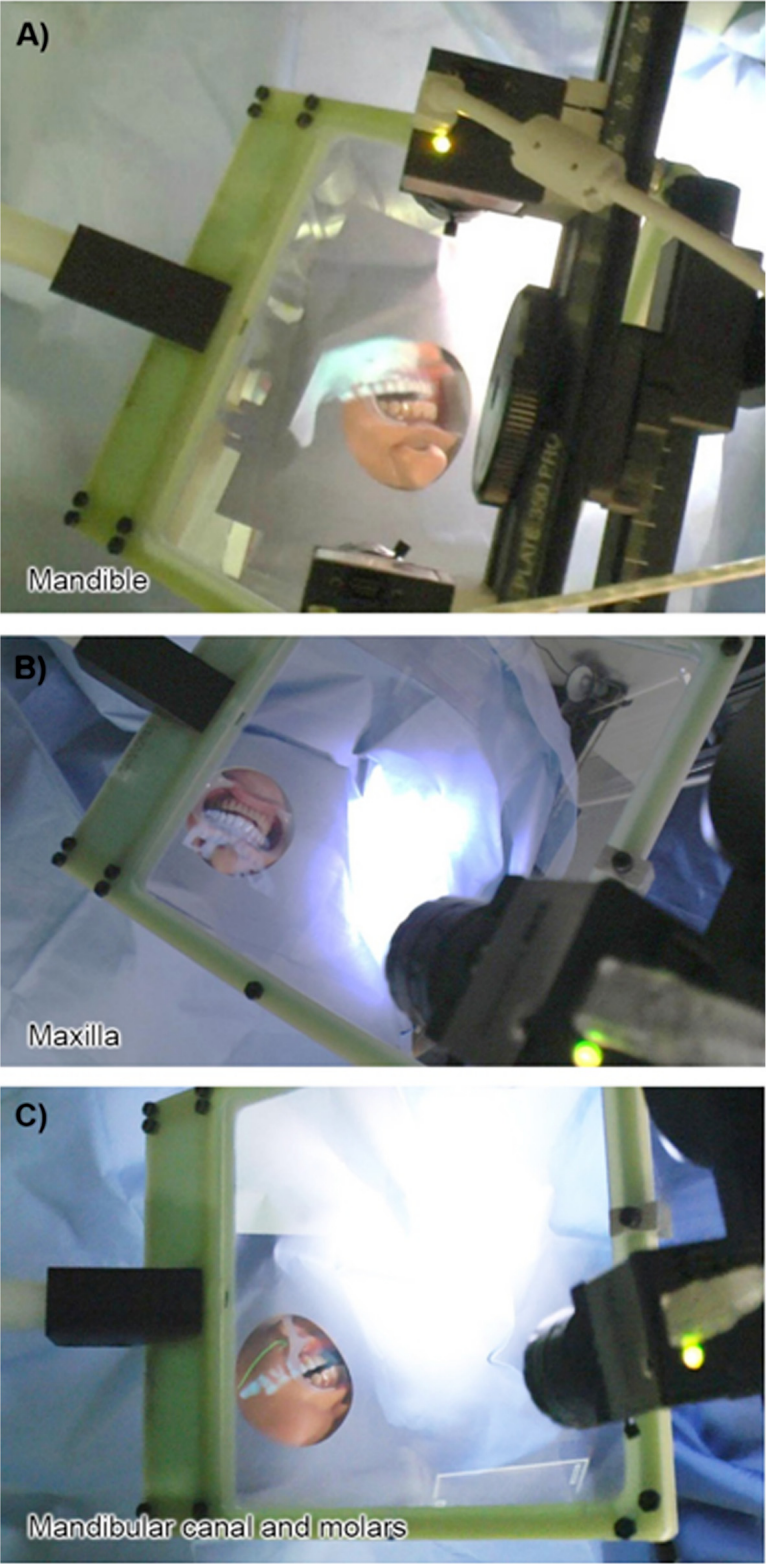
The IV images are overlaid on the surgical site. This included the **a** mandible, **b** maxilla overlaid on the surgical site, and **c** visualization of the mandibular canal, tooth root, and impacted third molar

**Fig. 5:**
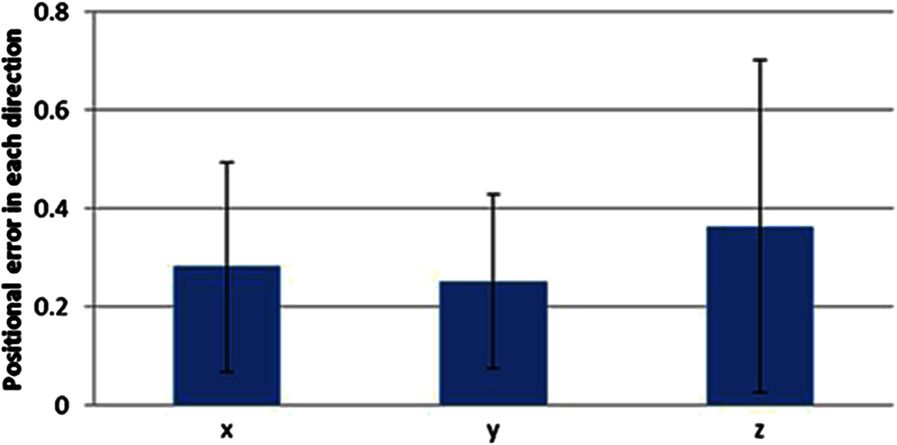
Positional errors along different axes (*x*, *y*, and *z*)

The calibrated images of the setup based on five feature points and the resulting 3D-IV image were displayed in real space as shown in Fig. 2a and 2b, respectively, which were then recognized and matched by our automatic measurement method using the stereo camera (Fig. 2c); extraction of characteristic points is shown in Fig. 3a. Registration of the 3D-IV and subject images was performed, wherein the contours were automatically detected, their outline generated and both were matched using the stereo camera (Fig. 3b). The 3D reconstruction of the teeth contours in the stereo camera after image matching is shown in Fig. 3c. By automatically detecting the feature points of the teeth, complete automatic registration was possible (Fig. 3b and 3c). Therefore, this system allowed real-time patient-image registration through tracking of teeth contours and image matching with the pre-operative model. The 3D-CT images of the mandible and maxilla in real space obtained using AR technology are shown in Fig. 4a and 4b (Additional files 1 and 2). Furthermore, CT data were displayed in real space as high-accuracy stereoscopic images with the teeth as landmarks for capturing information regarding the position of structures, thus negating the need for markers. The mandibular canal, tooth root and impacted third molar could also be visualized in the 3D-IV image (Fig. 4c; Additional file 3). The actual accuracy of the camera system was computed to be 0.096 mm along the XY axis and 0.403 mm along the Z axis (depth); the accuracy limit in positioning was theoretically calculated to be 0.425 mm. The error component in each direction is shown in Fig. 5. The mean (SD) error between the IV image and object was 0.28 (0.21) mm along the X axis, 0.25 (0.17) mm along the Y axis and 0.36 (0.33) mm along the Z axis.

The current study evaluated the feasibility of using a combination of AR and stereo vision technologies to project IV images obtained from preoperative CT data onto the actual surgical site during real time and automatic markerless registration, respectively, in a clinical setting on a volunteer. The existing methods using this type of system is so far done on phantom models. Here we have successfully done the markerless registration of patient image on a patient volunteer in a clinical setup for the first time. So there is no other similar study for comparison.

## Discussion

Dental surgery requires highly precise operations, with surgical targets often hidden by surrounding structures that must remain undamaged during the procedure. The use of AR system may provide a solution to address challenges presented in routine surgical practice.

The current study strategically simplified and improved the application of AR in OMS. The study used region-specific 3D-CT images displayed in real space with high accuracy and depth perception by using markerless registration through stereo vision. The study also used 320-row ADCT to provide a larger number of detector rows, and a single rotation of the gantry obtains 320 slices of CT images for a 16 cm volume area without a helical scan. Traditional methods of registration include an external registration frame or marker frames with a screw, [13, 24, 27] which are fraught with errors and also restrict the operating space. Furthermore, registrations for soft tissues are associated with low accuracy [28]. Zhu and colleagues [29] used an occlusal splint for registration in mandibular angle oblique-split osteotomy with good results; however, the system could not be used in edentulous patients and the markers were limited to the lower half of the face. A study using markerless registration showed variations between three different methods based on anatomic landmarks such as the zygoma, sinus posterior wall, molar alveolar, premolar alveolar, lateral nasal aperture and the infra-orbital areas that were used during navigational surgery of the maxillary sinus [30]. The results of that study showed that although the use of skin adhesive markers and anatomic landmarks was noninvasive and practical, it had limited accuracy and was restricted to craniofacial surgery. In the current study, complete automatic registration was possible because of the use of the anatomic features of teeth; they are the only hard tissues externally exposed, which makes them useful targets for registration based on 3D image matching via stereo vision. We believe that the registration procedure used in the present study can be used in the anterior teeth as well as molars (both jaws), thus including the entire oral surgery site; the only requirement being registration of the teeth. The introduction of stereo cameras into the IV image-overlay system eliminated the use of an external optical tracker.

During navigation in image-guided surgery, since the surgeon cannot simultaneously look at the screen and the operative site, this limitation can cause surgical errors [24]. In the current study using AR and stereo vision technologies, the IV image could be accurately aligned with the preoperative patient model, as observed from both directions. An added benefit was the facility to observe 3D images when changing the viewing position from horizontal to vertical on the subject similarly to real space without the need for special glasses. Because the 3D-AR display in this system obviated the need to avert the operator’s eyes from the surgical field or change focus, necessary information could be read in real-time during surgery.

In a comparative study of two navigation systems using frames for registration, the accuracy varied from 0.5 to 4 mm [27]. The average registration error in the current system approximated the theoretical value and was reported as 0.63 ± 0.23 mm, which was much lower than that reported in the other studies [31, 32]. The measurement resolution capacity of the stereo camera system in the current study was 0.1 mm along the XY axis and 0.4 mm along the Z axis. A comparative tracking error analysis of five different optical tracking systems showed that their position-measuring accuracy, ranging from 0.1 to 0.4 mm [33], was considered highly acceptable, whereas the position-measuring accuracy in the current study was theoretically calculated as 0.425 mm using the stereo camera [34]. Because our extraction algorithm was accurate up to the sub-pixel level, planar coordinates of a 3D point could be computed accurately to within 0.1 mm, which was superior to that of an optical tracking system. However, as both registration and tracking were carried out using a stereo camera set, measurement error was one of the major sources of registration errors. It was anticipated that the TRE for the proximal’characteristic features’ used for registration would be less than that in the region more distal. The pre-and intra-operative contours of each part (anterior or left and right molars) could be easily extracted using the method shown in this study, and the pair near the surgical site should be used for patient-image registration. Thus, the location of the surgical site should determine the selection of anterior teeth or molar tracking for patient-image registration. The potential for error increases with increases in the number of steps in a procedure. Therefore, the possibility of segmentation errors in CT data and registration errors related to incorporating digital dental models in CT data cannot be completely ruled out. In the field of oral surgery, the complexity of the anatomical structures involved often makes it difficult to visualize the operating field site. Ability to grasp the 3D relationships between such structures through direct visualization promises to greatly facilitate surgical procedures. Furthermore, the actual clinical accuracy in terms of clinical outcomes will require assessment of this procedure in surgery-specific randomized controlled trials.

## Conclusion

In conclusion, this system, without the use of references and fiducial markers, displayed 3D-CT images in real space with high accuracy. The system provided real-time markerless registration and 3D image matching using stereo vision, which, combined with AR, could have significant clinical applications. The entire registration process took less than three seconds to complete and the time for computation was acceptable. To improve accuracy and processing speed, future research should aim to improve the resolution of cameras, create finer displays and increase the computational power of the computer systems. Depth resolution could be improved using a camera with a smaller pixel pitch. Since an improved highly precise system is required, a high-definition rear display or lens array is needed for the densification of pixels in AR display. The most important contribution of this study is that it proved the feasibility of the AR system with IV imaging for clinical use with a registration error of < 1 mm. Our registration frame provided a markerless registration and tracking of IV images and patient, thereby simplifying intraoperative tasks, increasing accuracy and reducing potential discomfort and inconvenience to the patient. Therefore, this modified AR technique with stereo vision has a significant potential for use in clinical applications. In a sense, we think that the method adopted here cannot be rigidly classified into either a methodology or validation paper. In this paper it was termed as methodology because so many aspects of the methods were needed to conduct this work. The repeatability of the method may be in question if it is done only on one patient. Therefore, future works need to include clinical trials of this technology in more number of patients to assess a multitude of potential applications and universalization of this technique.

## Additional files

Additional file 1:
**Supplementary videos 1.** Real-time computer-generated integral images of the mandible are overlaid on the surgical site. (mp4 2.37MB) Additional file 2:
**Supplementary videos 2.** Real-time computer-generated integral images of the maxilla are overlaid on the surgical site. (mp4 1.21MB) Additional file 3:
**Supplementary videos 3.** Real-time computer-generated integral images of the mandibular canal, tooth root, and impacted third molar are overlaid on the surgical site. (mp4 2.42MB)

## Abbreviations

AR: Augmented reality
OMS: Oral and maxillofacial surgeries
IV: Integral videography
GCP: Good clinical practice
ADCT: Conditions for the area detector CT
RP: Rapid prototyping
PC: Phase correlation
ROI: Regions of interest
CT: Computed tomography
TRE: Target registration error
SD: Standard deviation
